# Variation in Arterial Stiffness and Markers of Oxidative Stress in Patients with Type 2 Diabetes Mellitus from Different Ethnic Groups

**DOI:** 10.3390/antiox14070858

**Published:** 2025-07-14

**Authors:** Karima Zitouni, Mia Steyn, Joanna Lewis, Frank J. Kelly, Paul Cook, Kenneth A. Earle

**Affiliations:** 1 Institute of Infection and Immunity, City St George’s, University of London, London SW17 0RE, UK; 2 St George’s School of Health and Medical Sciences, City St George’s, University of London, London SW17 0RE, UK; 3 Thomas Addison Diabetes Unit, St George’s University Hospitals NHS Foundation Trust, London SW17 0QT, UK; mia.steyn@gstt.nhs.uk; 4 Population, Policy & Practice Department, University College London, Great Ormond St., Institute of Child Health, London WC1N 1EH, UK; joanna.lewis@ucl.ac.uk; 5 Environmental Research Group, Faculty of Medicine, School of Public Health, Imperial College London, London W12 0BZ, UK; frank.kelly@imperial.ac.uk; 6 Chemical Pathology and Metabolic Medicine, University Hospital Southampton NHS Foundation Trust, Southampton SO16 6YD, UK; paul.cook@uhs.nhs.uk

**Keywords:** type 2 diabetes mellitus, cardio-renal disease, vascular stiffness, oxidative stress, 8-hydroxy-2′-deoxyguanosine, antioxidant enzyme activity

## Abstract

Diabetes is the world’s leading cause of renal and premature cardiovascular disease. There are marked differences between groups of patients with different ethnicities in their susceptibility to diabetes and its renal and cardiovascular complications. Novel markers of developing diabetes complications are related to disturbances in oxidative metabolism. In this cross-sectional study, we measured the arterial stiffness in patients of differing ethnicities with type 2 diabetes mellitus and assessed the relationship of their ethnicity with systemic markers of oxidative stress. Patients from black, African and Caribbean, and Asian minor ethnic groups were studied, with white patients with T2DM (*n* = 170) without evidence of cardiovascular disease (CVD). The vascular stiffness was measured by infrared finger-photoplethysmography. The oxidative stress burden was assessed by measuring the urinary 8-hydroxy-2′-deoxyguanosine (8-OHdG), activities of plasma glutathione peroxidase (GPx-3), superoxide dismutase (SOD) activities, and concentration of selenium. The vascular stiffness and 8-OHdG were higher in the white than in the Black patients (9.68 m/s vs. 9.26 m/s, *p* = 0.021 and 292.8 ng/mL vs. 200.9 ng/mL, *p* = 0.0027, respectively). Meanwhile, the GPx-3 and SOD activities and selenium were lower in the white than in the Black patients (283.3 U/L vs. 440.4 U/L, *p* < 0.0001; 37.5 U/L vs. 75.6 U/L, *p* = 0.0007; and 1.14 vs. 1.28 µmol/L, *p* = 0.0001, respectively). In regression modelling, the 8-OHdG/creatinine ratio was an independent predictor of vascular stiffness in the white patient group (β = 0.23 m/s per unit increase in ln(8-OHdG/creatinine) [95% CI, 0.03 to 0.42]; *p* = 0.021) but not in the Black patient group (*p* = 0.29). Increased vascular stiffness, lower endogenous antioxidant defense, and greater levels of oxidative damage were found in patients of white ethnicity, which could contribute to the higher incidence of CVD compared with patients from Black minor ethnic groups with diabetic renal disease.

## 1. Introduction

Type 2 diabetes mellitus is a worldwide public health problem with long-term vascular complications, which largely account for the associated morbidity and mortality [1]. A longitudinal study from the United States found that the odds of experiencing cardiovascular disease (CVD) were between 27% and 49% lower in minor ethnic groups than in whites [2]. A study of over 40,000 records of deaths of patients with type 2 diabetes mellitus from the United Kingdom revealed that patients from the minor Indo-Asian and African-Caribbean ethnicities both had similar, lower adjusted risks for mortality from CVD than white patients [3].

Vascular stiffness is a recognized factor that affects the pulse pressure and is a predictor of CVD [4,5]. Reduced arterial compliance is associated with type 2 diabetes mellitus, hypertension, and obesity [6,7,8]. The Framingham Heart Study of 2232 individuals confirmed vascular stiffness to be a predictor of CVD and mortality in the general population, hypertensive patients, the elderly, and patients with end-stage renal disease [9]. Moreover, measures of vascular stiffness have been shown to relate to changes in the glomerular filtration rate [10,11].

Oxidative stress and inflammation cause structural and functional changes in the vessel wall [12]. The most studied lesion of free radical-mediated damage is the modification of guanosine at the C-8 site, which produces 8-hydroxy-2′-deoxyguanosine (8-OHdG), which is related to exposure to dys-glycaemia [13]. The level of urinary 8-OHdG is an index of whole-body oxidative damage to DNA [14]. These changes are considered to cause premature ageing of the vasculature and, therefore, exposure to differing levels of oxidative stress could explain the varied propensity to CVD in patients with diabetes [15].

In this study, we investigated the relationship between the 8-OHdG level and arterial stiffness in patients with type 2 diabetes who were at high risk of cardio-renal disease. We also assessed whether any differences in vascular stiffness existed between patients from minor, Black African and Caribbean, and South Asian ethnic groups together when compared with white patients.

## 2. Materials and Methods

Patients with type 2 diabetes mellitus at increased risk of progressive kidney disease were recruited to a single site from general practices in Southwest London (United Kingdom) from April 2013 to May 2016, as part of a baseline analysis (prior to randomisation) of the PREVENT-2 study (Trial Registration ISRCTN: 97358113) [16]. Written informed consent was obtained from all participants prior to inclusion in this study and ethical approval was granted by the National Health Service Ethics Research Committee. Briefly, type 2 diabetes mellitus was diagnosed according to World Health Organisation criteria. Patients were included if they had at least two of the following characteristics, a history of hypertension (three consecutive sitting blood pressure readings >140 systolic, diastolic 90 mmHg without treatment, or receiving treatment for known hypertension), urinary albumin/creatinine ratio >3 mg/mmol, or a family history of hypertension, cardiovascular disease, or ESRD occurring in a first-degree relative <65 years of age. Patients were defined as being white if they self-selected their ethnic origin as white British, white Irish, or other white group and from a minor, Black, and/or Asian group if they selected Black Caribbean, Black African, Black other, Indian, Pakistani, Bangladeshi, Chinese, or other Asian.

Patients were excluded if they had a history of cardiovascular disease—defined as having a clinical record of ischaemic heart disease (angina, myocardial infarction, coronary artery revascularization, and/or heart failure), peripheral vascular disease (intermittent claudication or peripheral artery revascularization), or cerebrovascular disease (transient ischaemic episodes or stroke), were participating in another study, had a history of malignancy or any other life threatening illness, were currently pregnancy, had significant renal impairment (estimated glomerular filtration [eGFR] < 45 mL/min 1.73 m^2^), or had nephrotic range urine protein excretion (total protein excretion rate >3 g/day or albumin/creatinine ratio >300). Smoking habits were ascertained by interviews and patients were classified as—former smoker, current smoker, and never smoked.

### 2.1. Clinical and Anthropometric Assessments

Height was measured in metres, weight in kilograms, and waist circumference in centimeters. Body mass index (BMI) was calculated as weight in kilograms divided by height in meters squared.

Sitting blood pressure was measured by digital oscillometry (Omron 705IT; Omron Healthcare Europe, Hoofddorp, The Netherlands) according to the guidance of the National Institutes of Health and Care Excellence. Mean arterial pressure (MAP) was calculated from the following formula: MAP = [Systolic blood pressure  +  (2 × Diastolic blood pressure)/3].

### 2.2. Vascular Stiffness Index

Pulse wave amplitude was analyzed using infrared finger plethysmography (PulseTrace PCA2; CareFusion UK 232 Ltd., Basingstoke, UK). The readings were made over 10 min in a temperature-controlled room by trained nursing staff [7]. The stiffness index (SI) was automatically computed using the following formula: SI (m/s) = height of subject (m)/ΔT (s), where ΔT represents the time between the first systolic peak and the inflection point in the waveform [17]. SI was calculated from the mean of three measurements. The intra coefficient of variation was 6.75%. Five patients in each ethnic group failed to have their SI assessed due to technical reasons.

### 2.3. Biochemical Measurements

Venous blood was sampled after a 12 h fast. Plasma creatinine was measured using an isotope dilution mass spectrometry reference measurement procedure. Renal function was calculated as eGFR corrected for body surface area obtained from the plasma creatinine using the Chronic Kidney Disease Epidemiology Collaboration (CKD-EPI) race-adjusted equation. Glycosylated haemoglobin (HbA_1c_) was measured by immunoturbidimetry (ADVIA^®^ 2400; Siemens Diagnostics, Erlangen, Germany). Total triglycerides and total- and high-density lipoprotein (HDL)-cholesterols were estimated using an enzymatic assay (Roche system 702 on Cobas 8000/702; Roche Diagnostics, Mannheim, Germany).

### 2.4. Markers of Oxidative Metabolism and Defence

Urinary 8-OHdG—a marker of oxidative DNA damage, plasma enzymatic antioxidants (glutathione peroxidase and superoxide dismutase), and plasma antioxidant vitamin E and selenium, the co-factor for the enzyme glutathione peroxidase-3 (GPx-3), were assessed.

**8-OHdG** was measured using a competitive assay using an ELISA kit according to the manufacturer’s instructions (abcam, Cambridge, UK). The assay utilizes an 8-hydroxy-2-deoxyguanosine-coated plate and a horseradish peroxidase-conjugated antibody for detection. The assay recognizes both free 8-OHdG and DNA-incorporated 8-OHdG. 8-OHdG excretion was reported as both absolute and relative to urinary creatinine.

**Glutathione peroxidase** (GPx-3) activity was measured using a coupled assay system. The oxidation of reduced glutathione was coupled to NADPH oxidation in a reaction catalyzed by glutathione reductase [16].

**Superoxide dismutase** (SOD) activity was measured in relation to the inhibition of the rate of nitro blue tetrazolium reduction in the presence of xanthine and xanthine oxidase, which yielded a purple color [16].

**Vitamin E,** as α-tocopherol, was quantified by high-performance liquid chromatography with ultraviolet detection using tocopherol acetate as internal standard. α-tocopherol concentration in the samples was calculated by relating their peak areas to that of the internal standard [18].

**Selenium** (Se) was analyzed by dynamic reaction cell-inductively coupled plasma mass spectrometry NexION 300D (Perkin-Elmer, Beaconsfield, UK). ^78^Se was measured using ammonia (0.5 mL/min) as the dynamic reaction cell gas to remove the argon dimer background [19]. Samples were run against a Sigma Serum calibration curve, which was spiked with different concentrations of selenium standard reference solution at 1000 ppm (Fisher Chemical, Loughborough, UK). In order to eliminate interferences and thus increase the sensitivity of the signal, the samples were diluted with 0.5% butan-1-ol [20]. Rhodium was used as the internal stand ard. Internal Quality Control material was used throughout the assay, and the observed values were 0.60 ± 0.04 µmol/L (target 0.55 µmol/L), 1.16 ± 0.10 mol/L (target 1.15 µmol/L), and 2.08 ± 0.11 µmol/L (target 2.10 µmol/L).

### 2.5. Statistical Analysis

Data analyses were conducted using R software version R 4.2.2. Demographic and clinical variables are presented as mean ±  standard deviation or median [interquartile ranges)]; categorical variables are presented as percentages. α-tocopherol was reported per unit of cholesterol due to the dependence of plasma tocopherol concentration on lipid levels [21] and expressed as micromoles per millimoles. Urinary 8-hydroxy-2′deoguanosine concentration (in nanograms per milliliter) was reported as absolute and normalized for urinary creatinine concentration and was expressed as nanograms per milligram of creatinine. Continuous variables were analyzed with parametric or non-parametric tests according to their distribution. χ^2^ test was used as appropriate for univariable analysis of categorical variables. Spearman rank correlations were used for the entire cohort to investigate the associations between 8-OHdG, GPx-3, and SOD activities.

To investigate the relationship between vascular stiffness, ethnicity, and oxidative stress, we built linear regression models to predict vascular stiffness using ethnicity, 8-OHdG/creatinine ratio, and independent variables known to be associated with CVD: GPx-3 activity, mean arterial pressure, HbA1c, BMI, sex, age, diabetes duration, and smoking. The latter was entered as a behavioral factor for which there is strong evidence of its relation to vascular disease and its relationship with DNA damage markers [22]. We used the natural logarithm of the vascular stiffness index as our dependent variable to achieve a less skewed distribution of residuals. The natural logarithm of the 8-OHdG/creatinine ratio was used as our measure of oxidative stress to avoid undue influence on the model of a few patients with a very high 8-OHdG/creatinine ratio. A “full” multivariable model was fitted that included all predictors. We specified a priori that the GPx-3, BMI, sex, and 8-OHdG/creatinine ratio should remain in the model, and the full model was reduced by stepwise backwards selection of the mean arterial pressure, age, smoking, Hb1Ac, and duration variables, with a threshold *p*-value for inclusion of 0.05. We also fitted univariable models for all the predictors in order to compare estimated regression coefficients and *p*-values.

Ring et al. reported that patients with type 2 diabetes mellitus had a mean vascular SI of 10.0 + 2.0 [23]. We calculated that 63 subjects in each of the white, minor, Black, and/or Asian ethnic groups would provide 80% power using a 2-tailed alpha of 5% to detect a 1 m/s difference in SI. Differences of 1 m/s increments are reported to have a significant independent effect on the mortality in patients with diabetes [24].

## 3. Results

### 3.1. Demographic, Anthropometric and Clinical

Our total study cohort (*n* = 170) was made up of 69 patients of white ethnicity and 101 patients of minor, Black and/or, Asian ethnicity. The clinical data showed that both groups were similar in terms of their chronological age, systolic and diastolic blood pressure, fasting total-, HDL- and LDL-cholesterols, eGFR, and ACR. However, the white patients had significantly higher BMI, waist circumference, HbA_1c_, and triglyceride values and were more than twice as likely to have a history of smoking tobacco (61% vs. 28%; *p* < 0.0001) compared with the minor, Black, and/or Asian patients. (Table 1).

### 3.2. Vascular Stiffness and Oxidative Stress Status

**Figure 1:**
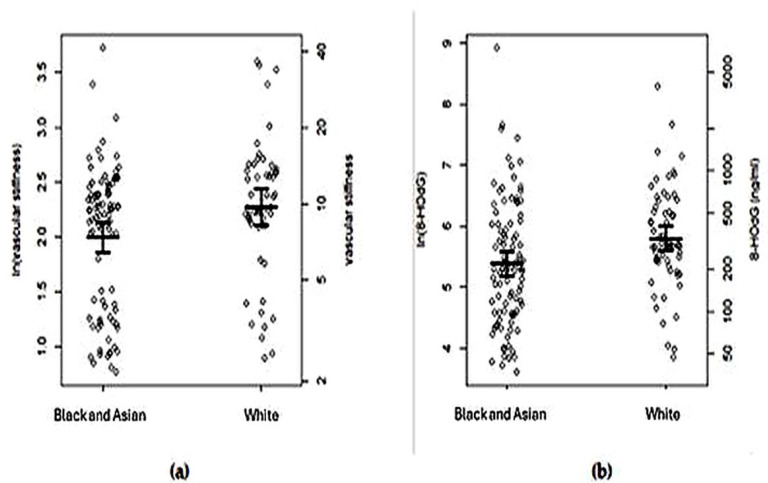
(**a**) Vascular stiffness index and (**b**) 8-hydroxy-2′-deoguanosine (8-OHdG) levels in white ethnicity (*n* = 64) and Black and/or Asian ethnicity (*n* = 96) patients with type 2 diabetes mellitus at high risk of cardio-renal disease. Circles show individual patient data, and black bars and error bars show the means and 95% CIs within each group.

Compared to the minor, Black, or Asian group, the patients of white ethnicity had significantly higher SI (median 9.68 m/s vs. 9.26 m/s; *p* = 0.021) and urinary 8-OHdG (median 292.8 ng/mL vs. 200.9 ng/mL; *p* = 0.0027) values (Figure 1).

The white ethnic group patients had lower glutathione peroxidase activity (median 283.3 U/L vs. 440.4 U/L; *p* < 0.0001), superoxide dismutase activity (median 37.5 U/L vs. 75.6 U/L; *p* = 0.0007), plasma selenium concentrations (median 1.14 µmol/L vs. 1.28 µmol/L; *p* = 0.0001), and α-tocopherol/total cholesterol ratios (median 7.9 µmol/mmol vs. 8.8 µmol/mmol; *p* = 0.02) compared with the minor, Black African and Caribbean, and Asian ethnic group of patients. In the whole cohort, the total 8-OHdG and 8-OHdG/creatinine had a negative relationship with GPx-3 activity (Spearman’s rho= −0.23, *p* = 0.0034 and rho= −0.17, *p* = 0.035, respectively).

The univariable and multivariable models for vascular stiffness are shown in Table 2. In the full multivariable model (all predictors included), there was evidence (*p* < 0.05) of an association between vascular stiffness and mean arterial pressure (estimated effect [95% CI] 0.017 [0.005, 0.030]; *p* = 0.0062), and between female sex (−0.27 [−0.50, −0.04]; *p* = 0.022) and ln (8-OHdG/creatinine ratio) in the white ethnic group of patients (0.22 [0.01, 0.43]; *p* = 0.044) only. In the reduced multivariable model (selected predictors only), there was evidence of an association between vascular stiffness and the same predictors: mean arterial pressure (0.021 [0.010, 0.031]; *p* = 0.0002), female sex (−0.23 [−0.44, −0.01]; *p* = 0.041) and ln (8-OHdG/creatinine ratio) in the white ethnic group of patients only (0.23 [0.03, 0.42]; *p* = 0.021). There was limited or no evidence that the fit of the reduced multivariable model was improved by re-introducing age (*p* = 0.11), smoking (*p* = 0.83), HbA1c (*p* = 0.31), diabetes duration (*p* = 0.10), or BMI (*p* = 0.052).

**Figure 2:**
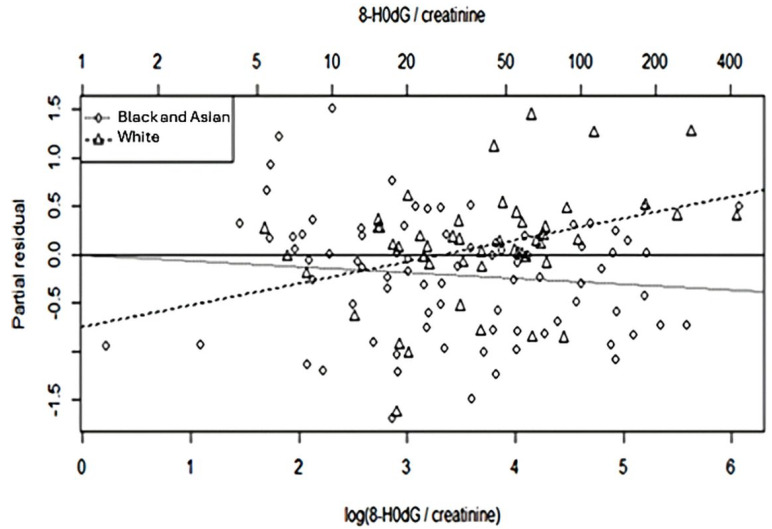
Partial residuals from the reduced multivariable model, accounting for mean arterial pressure, GPx-3, and sex but not ethnicity or ln (8-OHdG/creatinine). Diamonds represent Black and Asian patients and triangles represent white patients. The dotted and dashed lines represent the effect of ln (8-OHdG/creatinine) in Black and Asian patients and white patients, respectively. The solid horizontal line represents the zero residual reference.

The effect sizes and *p*-values were similar between the univariable and full multivariable models for mean arterial pressure, smoking, HbA1c, diabetes duration, GPx-3 activity, sex, and ln (8-OHdG/creatinine ratio) in both ethnic groups. Although the *p*-values for age differed, the confidence intervals estimated in the two models overlapped considerably (Table 2). The partial residuals for the reduced multivariable model, accounting for mean arterial pressure, GPx-3 and sex, were plotted against ln (8-OHdG/creatinine), which shows the difference in effect of the ln (8-OHdG/creatinine ratio) between minor, Black, and Asian patients and white patients (Figure 2).

## 4. Discussion

Our data show that the patients of white ethnicity with T2DM who are at high risk of cardio-renal complications have higher levels of 8-OHdG and arterial stiffness and lower, endogenous antioxidant defense when compared with patients of Black African and Caribbean and Asian ethnicity. These findings suggest that higher levels of oxidative stress contribute to the observed higher rates of CVD in some patients with T2DM.

Oxidative stress plays an important role in the development of diabetic vascular complications [25]; however, the evolution of kidney disease itself also confers a substantial increase in the risk of cardiovascular disease [26]. Therefore, gaining insight into the pattern of redox status with biomarkers in early nephropathy helps in understanding the evolution of CVD risk, from which these patients die prematurely.

Our findings are consistent with previous research showing that, among non-diabetic individuals, increased systolic blood pressure reactivity was more strongly associated with elevated 8-OHdG concentrations in Caucasian compared to African-American participants [27]. Together, these data suggest that the treatment of hyperglycaemia in diabetes may not address defects and differences in redox metabolism. Recent trials of therapies for T2DM have demonstrated significant cardiovascular, renal, and anti-cancer benefits that appear to be independent of their glucose-lowering effects [28,29].

In the diabetes milieu, hyperglycaemia induces excessive cellular free radical production. Higher oxidative glucose metabolism increases the mitochondrial production of superoxide radicals, which are converted to oxygen and hydrogen peroxide by SOD [30]. The endogenous antioxidant enzyme GPx-3 is one of the first to be activated in response to reactive oxygen, which converts it to harmless hydrogen peroxide and water [31]. When the GPx-3 activity is insufficient, hydrogen peroxide will be converted into the highly reactive hydroxyl radical due to the Fenton reaction attacking DNA, which yields 8-OHdG [32].

Smoking is a major determinant of the development of atherosclerotic vascular disease. Niu et al. reported that exposure of the cardiomyocytes to cigarette smoke increases the number of reactive oxygen species, depletes antioxidants, activates NF-κB, stimulates the production of inflammatory cytokine, and induces iNOS expression [12]. A systematic review of 14 studies that included epigenome-wide association studies and gene-specific methylation studies identified 1460 smoking-associated CpG sites in whole-blood DNA [22]. Moreover, a recent meta-analysis confirmed that healthy smokers have a significantly greater concentration of urinary 8-OHdG [33]. In our study, the relationship between 8-OHdG and vascular stiffness persisted after adjusting for smoking history, which supporting the hypothesis that factors that impair oxidative defense are associated with arterial stiffness.

A relative decrease in the plasma GPx-3, SOD activity, and selenium levels is indicative of diminished anti-oxidative capacity and an increase in oxidative stress. Studies have shown that increased oxidative stress causes endothelial dysfunction and vascular inflammation. Consequently, smooth muscle cell proliferation, the increased accumulation of elastin and collagen with extracellular matrix deposition, remodels and stiffens the arterial wall [34,35].

Elevated urinary 8-OHdG excretion has been reported in patients with T2DM with macrovascular and microvascular complications [36,37,38,39,40]. These reports confirm that urinary 8-OHdG is associated with vasculopathy. Together with our findings, these studies suggest that the measurement of 8-OHdG and a vascular stiffness index could help to discriminate those patients with T2DM who are at higher risk of developing both renal dysfunction and CVD.

This study is the first to report differences by ethnic group in vascular stiffness and 8-OHdG in a well characterized cohort of patients with T2DM who are at high risk of cardio-renal disease. It provides new insight into the complexity of cardio-renal risk. Our findings suggest that there may be opportunities to assess higher risk groups for cardiovascular disease early in the history of diabetes before the development of renal dysfunction, which augments vascular stiffness. The observed differences in vascular stiffness, which is greater in Black age, gender, and socioeconomically matched children compared to those of white descent, support our findings [41]. The host, behavioral, and/or environmental factors that cause these changes in vascular function in early and later life need to be explored.

Our assessment of vascular stiffness is consistent with that of other authors who have used the technique of carotid-pulse wave velocity measurement to show that increased vascular stiffness precedes a decline in glomerular filtration in patients at high-risk of kidney disease [27]. Moreover, the photoplethysmography stiffness index measures correlate well with derivatives such as the augmentation index from the carotid pulse wave velocity measurement in predicting the occurrence of cardiovascular events in high-risk patients and show a consistent pattern in ethnic differences [27]. The UK Biobank study with 169,613 participants showed that an arterial stiffness index—measured using photoplethysmography—above the cohort median was associated with a significantly increased hazard for CVD events and all-cause mortality after 2.8 and 6.1 years of follow-up, respectively, in comparison to values below the median [42]. The UK-based SABRE study reported that a derived mean arterial stiffness/elasticity index from carotid-femoral pulse wave velocity measurements (0.80 vs. 0.86 cPP/SV mmHg/mL; *p* < 0.001) was significantly lower in 214 patients of African-Caribbean descent compared with 589 patients of white ethnicity, respectively [43]. Ethnic differences in vascular stiffness in young adults may account for the aggressive nature of young-onset diabetes, which is rapidly increasing in frequency throughout the world [41].

There are limitations of our work, since we did not collect data on certain environmental factors including exposure to air pollution, alcohol intake, exercise pattern, and nutrient intake, which may affect 8-HOdG levels [44,45]. Our cohort was not large enough to examine the characteristics and obvious heterogeneity of smaller, specific ethnic groups within the broad categories, although this approach has strongly signaled the importance of ethnicity as a factor in disease phenotype [2]. This study did not include a matched non-diabetic control that could have offered additional insights into these associations in our studied population; however, other studies have shown a similar picture to ours in non-diabetic individuals [27]. Whether urinary 8-OHdG and arterial stiffness is a useful marker for the evolution of cardio-renal disease in T2DM awaits prospective studies with clinical outcomes, for which these data provides good evidence that needs further exploration.

## Figures and Tables

**Table 1:** Baseline demographic, anthropometric, clinical, and biochemical characteristics of patients with type 2 diabetes mellitus according to ethnic group.

Clinical Variables	Missing Data White/Minor, Black, or Asian	White (*n* = 69)	Minor, Black, or Asian (*n* = 101)
Age (years)	0/0	61.0 ± 7.93	60.52 ± 7.56
Diabetes duration (years)	1/7	8.0 (3.3–12.0)	9.0 (5.0–17.3) *
Gender: Male (%)	0/0	60.9	43.6 *
Smoking history: Yes/No/former-smoker (%)	3/2	15/39/45	4.0/72/24 ***
Retinopathy (%) †	19/17	40.0	43
Body mass Index (Kg/m^2^)	1/0	32.0 ± 5.4	29.4 ± 6.2 **
Systolic blood pressure (mmHg)	1/2	142)(133–151)	136 (128–151) *
Diastolic blood pressure (mmHg)	1/2	81 (77–87)	82 (87–89) *
ean Arterial pressure (mmHg)	1/2	102.5 ± 9.0	100.8 ± 10.1
HbA_1c_ (mmol/mol)	1/2	49 (44–59)	55 (48–67) **
HbA_1c_ (%)	1/2	6.6 (6.1–7.6)	7.2 (6.5–8.3) **
Total Cholesterol (mmol/L)	0/1	4.2 ± 0.9	4.0 ± 0.8
Triglycerides (mmol/L)	0/1	1.5 (1.0–2.1)	1.3 (0.9–1.7) *
HDL-Cholesterol (mmol/L)	0/1	1.27 ± 0.34	1.26 ± 0.35
LDL-Cholesterol (mmol/L)	2/1	2.1 ± 0.8	2.1 ± 0.6
eGFR CKD-EPI (mL min^−1^1.73 m^−2^)	0/1	93 (80–102)	92 (80–100)
Urinary Albumin/creatinine ratio (mg/mol)	0/5	0.8 (0.0–3.3)	0.8 (0.0–3.2)
Glutathione peroxidase activity (U/L)	2/3	289.4 ± 102.0	413.1 ± 98.52 ****
Superoxide dismutase activity (U/L)	1/3	37.5 (1.0–72.5)	75.6 (20.3–118.8) ***
Selenium (µmol/L)	1/2	1.16 ± 0.27	1.32 ± 0.24 ****
α-Tocopherol: total cholesterol (µmol/mmol)	1/3	8.6 ± 3.6	9.0 ± 2.1

Data are shown as mean ± SD, median (interquartile range), or percentage. * *p* ≤ 0.05, ** *p* ≤ 0.01, *** *p* ≤ 0.001, and **** *p* ≤ 0.0001. † retinopathy denotes the presence of background, pre-proliferative, or proliferative retinopathy. HbA_1_c indicates glycosylated haemoglobin type A1C; and GFR, glomerular filtration rate.

**Table 2:** Linear regression models for the association between ln (vascular stiffness) and predictors associated with cardiovascular disease and oxidative stress. Different numbers of patients were included in different models, due to missing data. One hundred and twenty-one patients were included in the full multivariable model, and 132 in the reduced multivariable model. The number of patients included in each univariable model is shown in the column headed “predictor”. In this model, ethnicity is an “intercept” term without a clinical interpretation, so the effect associated with it is not given in the table.

		Univariable Models (Unadjusted Effects)		Full Multivariable Model (*n* = 121) Adjusted R-Squared: 0.21		Reduced Multivariable Model (*n* = 132) Adjusted R-Squared: 0.19	
Predictor		Estimate (95% CI)	*p*-value	Estimate (95% CI)	*p*	Estimate (95% CI)	*p*
Mean arterial blood pressure (mmHg) (*n* = 142)		0.0213 (0.0105, 0.0321)	0.0002	0.0174 (0.0051, 0.0296)	0.0060	0.0205 (0.0100, 0.0311)	0.0002
Age (years) (*n* = 144)		−0.0157 (−0.0297, −0.0017)	0.028	−0.00463 (−0.01961, 0.01035)	0.54		
Smoking (current smoker) (*n* = 131)		0.0598 (−0.3509, 0.4706)	0.77	−0.114 (−0.571, 0.343)	0.62		
HbA_1c_ (mmol/mol) (*n* = 142)		0.000685 (−0.005577, 0.006947)	0.83	0.00452 (−0.00174, 0.01078)	0.16		
Diabetes duration(years) (*n* = 137)		−0.0115 (−0.0251, 0.0021)	0.096	−0.0115 (−0.0252, 0.0021)	0.096		
GPx-3 activity (U/L) (*n* = 141)		−0.000600 (−0.001590, 0.000389)	0.23	0.000237 (−0.000892, 0.001366)	0.68	0.000223 (−0.000844, 0.001290)	0.68
BMI (Kg/m^2^) (*n* = 144)		0.0317 (0.0148, 0.0485)	0.0003	0.0138 (−0.0050, 0.0325)	0.15		
Sex (female) (*n* = 144)		−0.233 (−0.447, −0.019)	0.033	−0.269 (−0.499, −0.039)	0.022	−0.225 (−0.441, −0.010)	0.041
Ln (8-OHdG/creatinine) (*n* = 134)	Minor, Black and Asian ethnicity	−0.101 (−0.220, 0.018)	0.095	−0.0900 (−0.2111, 0.0310)	0.14	−0.0610 (−0.1750, 0.0529)	0.29
	White ethnicity	0.201 (0.000, 0.402)	0.050	0.219 (0.006, 0.433)	0.044	0.226 (0.035, 0.418)	0.021

## Data Availability

The data that support the findings of this study are available from the corresponding author upon reasonable request.
